# Molecular weight-based profiling of sperm proteins and their association with semen quality in Kokok Balenggek roosters

**DOI:** 10.5455/javar.2025.l1003

**Published:** 2025-12-30

**Authors:** Jaswandi Jaswandi, Harif Gusdinal, Tulus Maulana, Ekayanti Mulyawati Kaiin, Rusfidra Rusfidra, Ananda Ananda

**Affiliations:** 1 Department of Animal Production Technology, Faculty of Animal Science, Universitas Andalas, Padang, Indonesia; 2 Research Center for Applied Zoology, National Research and Innovation Agency, Cibinong, Indonesia

**Keywords:** Kokok Balenggek rooster, Molecular weight, Semen quality, Sperm proteins, SDS-PAGE

## Abstract

**Objective:**

The study characterized the sperm proteins of Kokok Balenggek roosters based on molecular weight patterns to investigate the relationship between sperm composition and semen quality.

**Materials and Methods:**

Semen from 15 Kokok Balenggek roosters was collected and tested for standard quality indicators, including volume, motility, viability, abnormalities, and plasma membrane integrity (PMI). The protein content was measured, and the sperm protein profile was established by applying SDS-PAGE on a 10% polyacrylamide gel. In the next phase, the statistical correlation between the indicators of semen quality and the total protein levels, as well as the protein fractions defined by the molecular weight, was determined.

**Results:**

Through the SDS-PAGE analysis, a total of 46 protein bands with molecular weights ranging from 8 to ≥ 245 kDa were identified in KBR semen. The average volume of fresh semen was 597.67 ± 325.20 µl, the motility was 80.78% ± 12.92%, the viability was 87.56% ± 5.33%, the abnormality was 10.60% ± 2.30%, and the PMI was 85.06% ± 5.37%. There was a forceful positive correlation between the number of protein bands and sperm motility (*r* = 0.91, *p* < 0.01). High molecular weight proteins (≥ 218–247 kDa) showed negative associations with motility and viability, while low molecular weight proteins (< 30–40 kDa) were linked to sperm quality parameters in a positive way. The previous studies propose that these molecular weight ranges may be indicative of BSP proteins (~28–32 kDa), glycoproteins (~40–42 kDa), HSP70/HSP90 (~60–75 kDa), SPAG6 (~72 kDa), and fibronectin or larger heat shock proteins (~200–250 kDa), among others.

**Conclusion:**

The evaluation of KBR sperm proteins through their molecular weight profiles may yield initial clues to the potential semen quality markers, which is one of the main limitations of current research.

## Introduction

In poultry, the quality of semen and the efficiency of reproduction are the two primary factors that directly influence the breeding outcomes, such as fertility, productivity, and the preservation of genetic diversity, among others. For male chickens, evaluation of semen typically involves assessing plasma membrane integrity (PMI), motility, morphological abnormalities, and viability, all of which are closely related to the fertilization potential of females [1]. Of the semen quality parameters, sperm motility is the most important, as it indicates the sperm’s ability to reach the oocyte within the female reproductive tract. It is essential to keep the sperm alive and, at the same time, maintain the membrane integrity if the fertilization potential of the functional spermatozoa is to be preserved. On the other hand, changes in morphology may severely hinder reproductive performance [2–4].

The use of artificial insemination in studies with Kokok Balenggek roosters (KBRs) has revealed that semen quality is the primary characteristic responsible for determining both fertility and hatchability[5,6].

In the primary semen assays, which are predominant, still considerable disadvantages that could lead to unfavorable results. Using a microscope to assess the movement of sperm, their concentration, and their shape provides the researcher with a basic description of the condition; however, this alone is not enough to give a complete picture of the sperm's functional capacity. As a result, some males of bird species, such as chickens, which exhibit normal semen characteristics, may still be sterile due to undetected molecular abnormalities during routine analyses [7,8].

Furthermore, the use of manual microscopy may lead to subjective interpretation and observer variability, resulting in the dullness of both reproducibility and predictive reliability in semen assessments[9,10]. The use of technologies such as computer-assisted sperm analysis (CASA), flow cytometry, and fluorescence microscopy, among others, has significantly standardized sperm analysis[11. However, CASA still primarily focuses on evaluating kinematic traits and clarifying the mechanistic basis of sperm function, although it is costly and less accessible in low-resource settings, and remains restricted. Sperm dysfunction has been increasingly attributed to oxidative stress, which serves as a critical mediator influencing motility, viability, and fertilization ability[12–15]. This trend calls for molecular methods, which not only provide conventional assessments but also capture the underlying biological processes beyond these.

One of the factors that affects the variation in semen quality is its regulation by hormones. The correlation between fecal testosterone levels and semen parameters has been observed in different KBR phenotypes, supporting this finding [16]. To clarify the role of testosterone, it first supports the process of spermatogenesis, and secondly, it impacts the production of some sperm-associated proteins related to energy and capacitation. Antioxidant supplementation studies in avian semen provide strong evidence for the interplay of hormones, redox balance, and protein expression, especially when oxidative stress prevails [17,18].

Proteomic analysis enables the identification of proteins that are indispensable for essential reproductive functions, such as fertilization, motility, the acrosome reaction, and capacitation. The proteins identified as potential fertility biomarkers include proAKAP4, phosphatidylethanolamine-binding protein 4, mitochondrial enzymes, and heat shock proteins, which are significant in both avian and mammalian species [19–21]. Sperm proteins that exhibit different amounts in high-fertility and low-fertility males can serve as effective molecular markers in breeding programs for livestock species [22,23]. A recent study conducted by us defined sperm proteins and seminal plasma in relation to different Kokok Balenggek rooster phenotypes, revealing a significant diversity in protein expression within this local breed [24]. However, the protein molecular weights and their quality traits in KBRs have not been studied yet.

The technique of SDS-PAGE, which is a combination of sodium dodecyl sulfate and polyacrylamide gel electrophoresis, allows for the separation of proteins based on their molecular weight, which is a simple and cheap method that helps researchers to acknowledge the distinct color bands of the proteins that might indicate the quality of the semen [24–26].

Mass spectrometry, a resolution technique, is still considered a high-resolution method for the precise identification of species with little prior research; nevertheless, SDS-PAGE is still recognized as a worthy method for initial protein analysis 27]. In the case of birds, the sperm protein profiles linked to fertility traits have been revealed through the use of SDS-PAGE, thus providing users with insights that extend beyond traditional semen evaluation. The native chicken breeds are not only significant from a genetic perspective, but they also provide a solid foundation for sustainable poultry production in developing countries, primarily due to their ability to adapt, resistance to diseases, and socio-economic importance [28,29].

On the other hand, the distribution of commercial breeds and crossbreeding poses a threat to their genetic diversity and may lead to genetic erosion [30]. Conservation measures need to be implemented, and an extensive characterization of native breeds should be conducted to protect biodiversity and preserve traits that can be utilized for future breeding strategies. In Indonesia, the Kokok Balenggek rooster is distinguished by its unique crowing, adaptability, and reproductive power [31,32].

Research on KBR sperm has provided information about its quality parameters and storage conditions in various diluents, indicating that it is a suitable candidate for artificial insemination applications [33]. However, there has been limited molecular data available on the sperm protein composition.

The researchers of this study proposed that there is a connection between certain molecular weights of sperm proteins and quality traits of semen in KBRs. The research focused on characterizing sperm proteins through SDS-PAGE and their relationship to semen parameters. Thus, new molecular insights into the reproductive biology of roosters were gained, and at the same time, the conservation of native chicken breeds and their genetic improvement were supported.

## Materials and Methods

### Ethical approval

The Research Ethics Committee of the Faculty of Medicine, Universitas Andalas, Indonesia, reviewed and approved all procedures involving animals conducted in this study (Approval No: 473/UN.16.2/KEP-FK/2024) approval date: 3 December 2024.

### Experimental animals and management

This study utilized 15 KBR (*Gallus gallus domesticus*) aged 2–3 years, with an average body weight of 1.4 ± 0.3 kg. All roosters were healthy and free from any physical defects. Each bird was housed individually in a battery cage (45 × 50 × 60 cm) at the Poultry Unit, Universitas Andalas, Padang, Indonesia. The birds were fed a commercial breeder feed (ABS Crumble, JAPFA Comfeed Indonesia) supplemented with vitamins in the drinking water, provided ad libitum. Feeders and drinkers were maintained and cleaned daily, ensuring constant access to fresh water.

### Semen collection and evaluation

The semen of each rooster was collected three times at 3-day intervals using the dorso-abdominal massage method [34]; thus, a total of 45 ejaculates was produced. Collected ejaculates were placed in sterile microtubes, labeled, and analyzed for macroscopic traits (consistency, volume, pH, color, and odor) as well as microscopic traits, which included PMI, mass movement, concentration, abnormality, motility, and viability, according to established protocols [35–38].

Sperm motility was quantified as the percentage of progressively motile sperm using a light microscope (Axiocam HRc, Carl Zeiss, Germany) at 400x magnification, while mass movement was scored from—(absent) to +++ (excellent). After semen dilution in 3% NaCl, sperm concentration was determined using a hemocytometer. Viability and abnormal morphology were examined through eosin–nigrosin staining, and PMI of 200 spermatozoa was assessed via the hypo-osmotic swelling test under phase-contrast microscopy.

### Protein extraction from sperm cells

Ejaculates with motility of 70% or more were subjected to centrifugation after three consecutive washes with (phosphate-buffered saline) at 4°C to separate sperm cells from seminal plasma. The sperm pellets from each collection were stored at –20°C until needed. At the end of the collection period, three frozen sperm pellets from each rooster were thawed and combined to create a sample that represents each individual, thereby yielding 15 final sperm samples for protein analysis. Sperm pellets were preserved using the freezing method at −20°C without any cryoprotectants until pooling, simulating the typical conditions of semen storage prior to artificial insemination research processing, while also acknowledging that freezing-thawing could affect certain membrane proteins. Using PRO-PREP™ Protein Extraction Solution (iNtRON Biotechnology, Seoul, Korea), samples were resuspended, homogenized, and incubated at –20°C for 20 min, followed by centrifugation at 13,000 rpm for 5 min at 4°C; the supernatant enriched with extracted proteins was harvested and preserved at –20°C until further use [37,38].

#### Protein quantification

Using a spectrophotometer (UV-1800, Shimadzu, Kyoto, Japan), absorbance at 595 nm was measured after determining the total protein concentration via the Bradford assay, with bovine serum albumin (BSA; Sigma-Aldrich, St. Louis, MO, USA) used as the reference standard [35].

#### SDS-PAGE and molecular weight estimation

**Figure 1.**
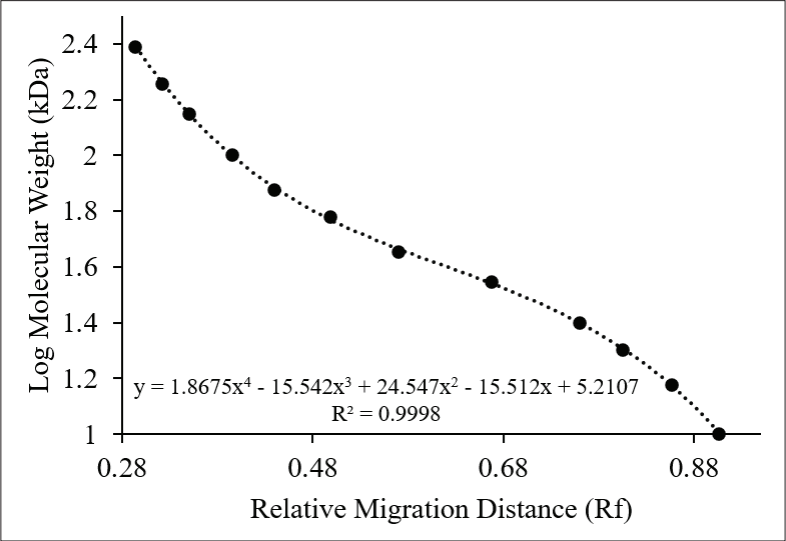
Standard calibration curve for SDS-PAGE. Protein markers (PM2700, SMOBIO, Taiwan) were used to correlate the relative migration distance (Rf) with the logarithm of molecular weight (kDa). Using ImageJ software, the molecular weights of the sperm protein bands in KBr were calculated based on a polynomial regression equation (R² = 0.9998), confirming the high precision of the estimation.

Using SDS-PAGE, proteins extracted from spermatozoa were resolved on precast 10% polyacrylamide gels (Q-PAGE™, SMOBIO, Taiwan). The 10% gel concentration was selected because it provides reliable resolution for proteins in the 10–245 kDa range, which was the primary focus of this study. Equal amounts of protein (approximately 25 µg in 20 µl per lane) were loaded to ensure comparable band intensity across samples, based on normalization of protein concentrations obtained from the Bradford assay. After denaturing the sample proteins at 70°C for 10 min in NuPAGE™ LDS Sample Buffer (Thermo Fisher Scientific, Waltham, MA, USA), they were loaded into gel wells along with a prestained molecular weight marker (ExcelBand™, SMOBIO, Hsinchu, Taiwan). Electrophoresis was carried out for 90 min at 110 V using a BIO-RAD vertical system (Hercules, CA, USA). Following overnight staining with Fast Coomassie Blue (Elabscience, Wuhan, China) and rinsing in distilled water, the gels were visualized using the iBright 1,500 imaging system (Thermo Fisher Scientific, Waltham, MA, USA). The relative migration of each protein band was quantified using ImageJ (NIH, Bethesda, MD, USA) and compared to the molecular weight standards. Protein molecular weights were subsequently calculated with a polynomial regression formula:

y = 1.8675x⁴ − 15.542x³ + 24.547x² − 15.512x + 5.2107; R² = 0.9998

where y corresponds to log molecular weight (kDa) and x represents the measured Rf. The regression curve was generated specifically from the marker bands (Fig. 1).

### Data analysis

The various traits of semen quality were initially summarized with descriptive statistics defined as mean ± SD. To detect significant differences between single roosters, a one-way ANOVA was performed, followed by Duncan's multiple range test to separate means where significant differences were found (*p* < 0.05). A Pearson's correlation test, conducted using SPSS version 25.0 (IBM Corp., Armonk, NY, USA), was utilized to investigate the relationships among semen quality factors, protein concentration, and protein bands of different molecular weights. The consideration of effect sizes was also made to enhance the statistical interpretation [38].

### Results and Discussion

#### Quality of fresh semen from Kokok Balenggek roosters

The average semen volume of KBR was 597.67 ± 325.20 µl, as shown in Table 1, which presents the characteristics of fresh semen. This value exhibits high variability, with the standard deviation accounting for almost 54% of the mean. This variability indicates the possibility of outliers or individual physiological differences among roosters; if not using statistical methods such as outlier diagnostics, this may skew the interpretation of correlations. However, the values observed were within the wide range reported in other indigenous breeds, such as 0.05–1.0 ml in Castellana chickens [39], 0.22–0.36 ml in Thai natives [40], and ~0.6 ml in Tanzanian Horasi ecotypes. The semen was characterized by a neutral to slightly alkaline environment, having a mean pH of 7.11 ± 0.24, which was considered optimal for sperm viability. The averages of the motility, viability, and PMI were 80.78% ± 12.92%, 87.56% ± 5.33%, and 85.06% ± 5.37%, respectively, while the sperm abnormality was 10.60% ± 2.30%, which is quite low. These characteristics exceed the threshold for artificial insemination, where a motility of 70% is required for acceptable fertility [41]. In Kedu, Sentul, and Kampung chickens, similar values have been reported with motility of 75%–85% and viability of nearly 90% [42,43].

The variation among individuals (Table 2) is anticipated in native breeds, wherein genetic diversity and environmental adaptation promote phenotypic variation [44]. Climatic and environmental factors are of great importance, as high temperatures and humidity reduce motility and sperm concentration in tropical birds 45]. These factors underscore the need for multiple sampling points rather than single-point sampling in semen studies. Moreover, the quality of the semen must be assessed in conjunction with the management practices: over-restraining, inefficient collection techniques, or stress during handling may reduce the quality of the ejaculate without affecting the inherent sperm characteristics. An increase in corticosterone due to stress has been demonstrated to affect testicular function and sperm physiology in birds and mammals, indicating the need to consider behavioral and management factors when determining semen quality.

#### Protein concentration and SDS-PAGE profiles of sperm proteins

Table 2 illustrates that the concentration of sperm protein in Kokok Balenggek male chickens varied between 0.22 and 0.99 mg/ml, with an average value of 0.66 ± 0.19 mg/ml. The nearly a five-fold difference in concentrations among males indicates a significant physiological variation among the birds. Such variations might be due to differences in testicular activity, spermatogenic efficiency, or sperm protein turnover. For instance, the intake of dietary antioxidants, the burden of oxidative stress, and the age of males have been associated with the level of sperm protein in poultry and livestock [14,23].

**Table 1.** Fresh semen characteristics of Kokok Balenggek roosters.

Semen evaluation parameters	Mean
Macroscopic characteristics	
Volume (µl)	597.67 ± 325.20
Odor	Rooster-specific
pH	7.11 ± 0.24
Color	Milky White
Consistency	Thick
Microscopic characteristics	
Mass Movement	++
Motility (%)	80.78 ± 12.92
Concentration (10⁹/ml)	1.99 ± 1.14
Viability (%)	87.56 ± 5.33
Abnormality (%)	10.60 ± 2.30
Plasma Membrane Integrity (%)	85.06 ± 5.37

**Table 2.** Individual semen quality and sperm protein concentration of Kokok Balenggek roosters.

Rooster ID	Sperm Motility (%)	Sperm Viability (%)	Sperm Abnormality (%)	Sperm PMI (%)	Sperm PC (mg/ml)
BR1	90.00 ± 0.00^a^	92.67 ± 0.76^a^	11.33 ± 0.76^a^	89.67 ± 0.29^a^	0.22
BR2	85.00 ± 5.00^abc^	89.00 ± 1.00^bcd^	9.00 ± 1.00^a^	87.33 ± 1.26^bc^	0.57
BR4	85.00 ± 0.00^abc^	85.67 ± 1.04^e^	10.50 ± 2.50^a^	83.33 ± 0.29^e^	0.81
BR5	86.67 ± 2.89^ab^	88.67 ± 0.29^bcd^	10.67 ± 1.76^a^	85.33 ± 0.29^cde^	0.99
BR6	71.67 ± 2.89^d^	88.50 ± 2.00^bcd^	9.00 ± 2.00^a^	86.33 ± 1.76^bcd^	0.25
BR7	86.67 ± 2.89^ab^	90.67 ± 1.26^b^	11.33 ± 2.25^a^	88.33 ± 1.26^ab^	0.72
BR8	81.67 ± 2.89^bc^	88.00 ± 0.50^cd^	10.33 ± 1.26^a^	86.00 ± 0.50^cd^	0.71
JK1	80.00 ± 0.00^c^	87.50 ± 1.50^de^	10.50 ± 3.00^a^	84.17 ± 1.26^de^	0.72
KN1	81.67 ± 2.89^bc^	88.33 ± 1.76^cd^	10.33 ± 0.76^a^	86.33 ± 1.26^bcd^	0.69
BL1	86.67 ± 2.89^ab^	89.50 ± 0.50^bcd^	9.67 ± 2.75^a^	87.00 ± 1.00^bc^	0.72
BR10	81.67 ± 2.89^bc^	88.00 ± 1.00^cd^	12.00 ± 1.00^a^	86.00 ± 1.50^cd^	0.66
JK2	86.67 ± 2.89^ab^	90.00 ± 0.50^bc^	9.33 ± 0.76^a^	86.50 ± 1.00^bc^	0.66
JK3	36.67 ± 5.77^e^	69.00 ± 1.50^f^	16.33 ± 1.76^b^	66.33 ± 1.76^f^	0.70
KN2	86.67 ± 2.89^ab^	88.17 ± 1.76^cd^	9.33 ± 1.26^a^	85.67 ± 1.76^cd^	0.72
BL2	85.00 ± 5.00^abc^	89.67 ± 0.29^bcd^	9.33 ± 0.76^a^	87.50 ± 1.00^bc^	0.73
Mean ± SD	80.78 ± 12.92	87.56 ± 5.33	10.60 ± 2.30	85.06 ± 5.37	0.66 ± 0.19

PMI = sperm plasma membrane integrity; PC = protein concentration; Means in a column with different superscripts a and b differ significantly (*p* < 0.05).

 Another factor that might influence sperm protein levels is the season. Chickens subjected to heat stress develop a different seminal protein profile, with an increased amount of chaperones and stress proteins [45]. Hence, such variations in sperm, indicated by changes in protein concentration, are considered biologically acceptable, and at the same time, they provide insight into the complexity of regulating the sperm proteome.

**Figure 2.**
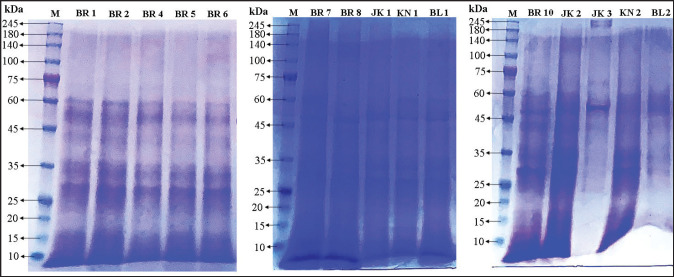
SDS-PAGE analysis of sperm protein profiles from Kokok Balenggek roosters. A 10% polyacrylamide gel was used to separate the protein bands. Lanes BR1–BL2 corresponded to individual Kokok Balenggek roosters, and PM2700 (SMOBIO, Taiwan) served as the molecular weight reference in the leftmost lane, ranging from 10 to 245 kDa. Gels were documented using the iBright imaging system (Thermo Fisher Scientific).

During the SDS-PAGE analysis, a total of 46 protein bands were detected with an approximate weight ranging from 10 kDa to 245 kDa, as depicted in Table 3 and Figure 2.

The main groups that were expressed the most consistently were from the middle weight ranges: 59–45 kDa (93.3% of the samples), 44–35 kDa (73.3%), and 34–25 kDa (73.3%). The mentioned ranges have been previously associated with sperm motility and fertilization in birds and mammals [26,46].

The low-molecular-weight proteins (~28–32 kDa) are probably those that belong to BSPhomologs, which are responsible for sperm-egg binding and capacitation [4], while the proteins in ~40–42 kDa are those glycoproteins that help in perivitelline penetration [48]. The proteins at the intermediate level (~60–75 kDa) are composed of HSP70/90 chaperones and SPAG6, with the former being the dominant ones in the flagellum's operation and the latter playing a role in resistance to stress [49]. Fibronectin and large heat shock proteins, for example, high-molecular-weight proteins exceeding 200 kDa, may be indicative of structural or stress-related processes; however, their high levels have been negatively associated with semen quality in several species [50]. The existence of the proteins in the sample, however, is still uncertain. SDS-PAGE simply gives a separation based on the approximate molecular weight, and without densitometry, Western blotting, or mass spectrometry, the identities of the candidate proteins cannot be confirmed. Therefore, the correlations stated here must be viewed as suggestive rather than conclusive. Rooster fertility will be a topic for which the specific proteins involved and their functional roles will be validated by future studies using LC-MS /MS or targeted immunodetection.

#### Correlations between protein profiles and semen quality traits

A correlation analysis conducted on the dataset from the experiment yielded three key findings (Table 4. First, a moderate correlation was observed between protein concentration and motility (*r* = 0.42); meanwhile, the correlations to viability (*r* = −0.199), PMI (*r* = −0.213), and abnormality (*r* = 0.103) were all of a weak nature. It follows that the total protein content supports motility, perhaps through energy metabolism, but does not have a direct impact on cell survival or morphology. In fact, motility is dependent on and supported by glycolytic and oxidative phosphorylation pathways that are activated by adenosine triphosphate (ATP) synthase, GAPDH, and other metabolic proteins [23,51]. On the other hand, viability is predominantly influenced by the level of oxidative stress, age, and environmental conditions [4,45]. The results suggest that it is crucial to exercise caution in not overemphasizing protein concentration as a predictor of fertility. Its moderate impact on motility is of biological importance but not enough to qualify it as a standalone biomarker.

**Table 3.** Distribution of sperm protein bands by molecular weight in Kokok Balenggek roosters.

MW (kDa)	Rooster ID															Protein Absence*
	BR1	BR2	BR4	BR5	BR6	BR7	BR8	JK1	KN1	BL1	BR10	JK2	JK3	KN2	BL2
≥ 245	−	−	−	−	−	−	−	−	−	−	−	−	+	−	−	1/15 (6,7)
244–180	−	−	−	−	−	−	−	−	−	−	−	−	+	−	−	1/15 (7,7)
179–140	−	−	−	−	−	−	−	−	−	−	−	−	−	−	−	0/15 (0)
139–100	−	−	−	−	−	−	−	−	−	−	−	−	−	−	−	0/15 (0)
99–75	−	−	−	−	−	−	−	−	−	−	−	−	−	−	−	0/15 (0)
74–60	+	−	−	−	−	+	−	−	−	−	−	−	−	−	−	2/15 (13,3)
59–45	+	+	+	+	+	+	+	+	+	+	+	+	+	−	+	14/15 (93,3)
44–35	+	−	−	+	−	−	−	−	−	−	+	+	−	−	−	4/15 (26,7)
34–25	+	+	+	+	+	+	−	+	+	+	+	−	−	+	−	11/15 (73,3)
24–20	−	−	−	−	−	−	−	−	−	−	−	−	−	−	−	0/15 (0)
19–15	−	−	−	−	−	−	−	−	−	−	−	−	−	−	−	0/15 (0)
14–10	−	−	−	−	−	−	−	−	−	−	−	−	−	−	+	1/15 (6,7)
≤ 9	+	+	+	+	+	+	+	+	+	+	+	+	−	−	−	12/15 (80)
Σ Bands	5	3	3	4	3	3	4	2	3	3	4	3	3	2	1	46/195 (0,3)

*= *n* (%); MW= molecular weight; + = protein expressed; − = protein non-expressed.

Second, the number of protein bands presented a very strong positive correlation with motility (*r* = 0.91), indicating that the diversity of the proteome is a supporting factor in sperm function. Roosters exhibiting a wider spectrum of protein expression were more often characterized by increased motility, which likely indicates that their metabolic and structural pathways are robust and exhibit some degree of overlap. Similar trends have been observed in indigenous poultry and livestock, where protein diversity has been associated with predicting higher fertility outcomes [52]. Nevertheless, correlations with viability (*r* = 0.133), PMI (*r* = 0.100), and abnormality (*r* = 0.314) were weak and statistically non-significant, thus emphasizing that traits other than motility are less reliant on proteomic variation and more controlled by intrinsic genetics or spermatogenic fidelity. The analysis of molecular weight categories, the third point, indicated that high molecular weight proteins (218–247 kDa) had a negative correlation with sperm motility, viability, and PMI counting, whereas they had a positive correlation with abnormality. This suggests that stress-related proteins being overexpressed may lead to impairment of sperm function. On the contrary, proteins in the range of 30–75 kDa, including HSP70/90, SPAG6, and glycolytic enzymes, were those for which semen quality was favorable throughout the study. The study results have categorized mid-range proteins as potential biomarker candidates, but the final identification is dependent on mass spectrometry validation. It should always be taken into account that these were purely associative findings and not causal ones. The non-triviality of the Type I error risk arises from the fact that no adjustments were made to multiple correlations tested at once. Moreover, the lack of replicates and the use of densitometry make the band quantification less robust. It is necessary to conduct further studies using regression modeling, mediation analysis, and a larger sample size to determine whether some proteins directly influence motility or if both are simply co-regulated by broader physiological processes.

#### Biological interpretation and integration with reproductive physiology

The mentioned relationships correspond to the broader reproductive mechanisms. Proteins with molecular weights of 30–75 kDa are functionally connected to energy metabolism and flagellar movement. GAPDH and ATP synthase are the entry points of the glycolytic and oxidative phosphorylation pathways, respectively, supplying the ATP necessary for motility. SPAG6 stabilizes the axonemal structure, while HSP70/90 facilitates protein folding and protects proteins from oxidative stress. Thus, the positive relationships between these proteins and motility signal the very well-acknowledged functional pathways (Table 5)

On the other hand, the negative correlations with high-MW proteins point towards a stress-related phenotype. The expression of large chaperones, such as HSP90A, has been associated with impaired sperm motility in bulls and infertility in poultry [50,53].

**Table 4.** Correlation coefficients (r) and *p*-values (*p*) between sperm protein concentration, number of protein bands, molecular weight of sperm proteins, and semen quality parameters in KBr.

Parameter / Protein MW (kDa)	Motility (*r,p*)	Viability (*r,p*)	Abnormality (*r,p*)	PMI (*r,p*)
Protein concentration (mg/ml)	0.042, 0.881	−0.199, 0.478	0.103, 0.715	−0.213, 0.446
Protein bands (*n*)	0.091, 0.746	0.133, 0.636	0.314, 0.254	0.100, 0.723
247 kDa	−0.943**, 0.000	−0.956**, 0.000	0.867**, 0.000	−0.958**, 0.000
218 kDa	−0.943**, 0.000	−0.956**, 0.000	0.867**, 0.000	−0.958**, 0.000
61 kDa	0.237, 0.394	0.311, 0.259	0.162, 0.563	0.297, 0.283
54 kDa	−0.075, 0.789	0.018, 0.949	0.144, 0.609	0.024, 0.932
42 kDa	0.263, 0.344	0.264, 0.341	0.079, 0.779	0.210, 0.452
31 kDa	0.403, 0.136	0.399, 0.141	−0.229, 0.412	0.408, 0.132
11 kDa	0.126, 0.655	0.032, 0.911	−0.192, 0.493	0.031, 0.911
8 kDa	0.435, 0.105	0.490, 0.063	−0.326, 0.236	0.487, 0.066

PMI=Plasma Membrane Integrity; *r* = Pearson correlation coeff. *p* = *p*-value (2-tailed).

**Significant correlations: **p < 0.05; *p* < 0.01.

**Table 5.** Candidate sperm proteins and their potential roles in semen quality of KBr.

Molecular weight (kDa)	Candidate protein	Reported function	Species studied	References
~28–32	BSP proteins (Binder of Sperm, e.g., BSP-A1/A2, BSP-A3)	Capacitation, sperm storage, sperm–egg binding	Bull, boar, suggested in poultry	[47,55]
~40–42	Glycoproteins (e.g., sperm–egg binding proteins)	Sperm activation, perivitelline layer penetration	Chicken, quail	[48]
~60–75	HSP70/HSP90 variants	Stress response, protein folding, sperm protection	Bull, chicken, human	[50,56]
~72	SPAG6 (Sperm-associated antigen 6)	Flagellar structure and function, motility regulation	Rooster, human	[49]
~200–250	Fibronectin, large HSPs	Structural support, sperm–oviduct interaction, capacitation	Chicken, mammal	[53,54]

Note: Although these candidate proteins have not all been experimentally confirmed in KBr, they are inferred from prior studies in poultry and mammals as potential functional equivalents that may play similar roles in sperm physiology.

At around 250 kDa, fibronectin is the protein that facilitates sperm-oviduct interactions. However, its change in expression may disrupt both capacitation and sperm storage [54]. These claims suggest that the mid-MW proteins, despite being mostly supportive of sperm function, might get excessively expressed and thus signal cellular stress or dysregulation.

The role of oxidative stress becomes very significant within this context. Reactive oxygen species deteriorate the membrane and weaken the DNA, leading to the death of the sperm and a decrease in the PMI even when motility is still at an acceptable level. Some of the proteins that have been tentatively identified, such as HSP90 and antioxidant enzymes, are all part of the defense mechanism against oxidative damage, thus connecting the proteomic profile to sperm survival pathways [12,15].

This raises an issue that has not been fully examined yet: the transition from motility to diverse protein expression is the primary reason for the latter's vulnerability to oxidative imbalance, which also explains the weak negative correlations with total protein concentration observed in this study. The breeding process not only has to take into account the psychological and behavioral factors. It is possible that the indigenous roosters that were subjected to the repeated collection of semen experienced the stress of handling, which in turn raised the levels of corticosterone and negatively affected the testicles. It has been known that stress can affect protein expression, regardless of fertility level, resulting in a misleading interpretation of the observed associations. Stress-mitigation strategies should be incorporated into breeding management, as they will help ensure that the proteomic biomarkers reflect the true reproductive potential and not just handling artifacts.

#### Limitations and future perspectives

This research is the first systematic evaluation of sperm protein profiles in Kokok Balenggek roosters; however, it is limited by a few factors. The high variation in semen traits, first of all, necessitates larger sample sizes and multiple measures to account for outliers and environmental effects in the case. Secondly, SDS-PAGE gives only approximate protein separation; without densitometry or replicates, quantification is limited, and protein identification remains speculative. The associations presented here are therefore provisional and require confirmation using LC-MS/MS or Western blotting with specific antibodies. Thirdly, multiple correlation testing increases the risk of Type I error. Although effect sizes like *r* = 0.91 are very persuasive, stronger inferences require statistical adjustments and scatter plot validation. The study, however, despite these limitations, has laid down the foundation that motility is the semen characteristic most consistently related to protein profiles, especially in the 30–75 kDa range. The future research is suggested to be carried out in the manner of (i) validating the candidate proteins through proteomics, (ii) incorporating oxidative stress markers and hormonal profiles, and (iii) examining protein expression influenced by season and management practice. Such strategies will enhance the mechanistic understanding of rooster fertility and, simultaneously, increase the applicability of proteomic markers for conservation and breeding programs.

### Conclusion

The first characterization of sperm protein profiles in the Kokok Balenggek roosters and their correlation with semen quality formed the basis of this study. The ejaculates presented high motility, viability, and plasma membrane integrity, while the SDS-PAGE analysis exhibited protein bands in the range of 30–75 kDa, which were positively correlated with motility. On the contrary, the high-molecular-weight proteins (> 200 kDa) were negatively correlated with semen quality traits. Thus, it can be inferred that mid-range proteins may contribute to reproductive performance, while large proteins may indicate stress-related processes. Nonetheless, the results need to be interpreted with caution, as the correlations are associative and not causal, and the identification of proteins by SDS-PAGE remains tentative without validation via LC-MS/MS or Western blotting. Additionally, differences between males and the potential effects of stress and environmental factors may obscure the observed relationships. In conclusion, the study provided a valuable basis for further research into the fertility of indigenous cocks. However, verifying the selected candidates and enhancing their use in breeding and conservation programs would necessitate the integration of state-of-the-art proteomic tools, oxidative stress markers, and seasonal aspects in future research.

### List of abbreviations

AI, artificial insemination; ANOVA, analysis of variance; ATP, adenosine triphosphate; BSP, binder of sperm protein; DNA, deoxyribonucleic acid; GAPDH, glyceraldehyde-3-phosphate dehydrogenase; HMW, high molecular weight; HOST, hypo-osmotic swelling test; HSP, heat shock protein; KBR, Kokok Balenggek rooster; LC-MS/MS, liquid chromatography–tandem mass spectrometry; MW, molecular weight; PBS, phosphate-buffered saline; PMI, plasma membrane integrity; SD, standard deviation; SDS-PAGE, sodium dodecyl sulfate–polyacrylamide gel electrophoresis; SPAG6, sperm-associated antigen 6; SPSS, statistical package for the social sciences.
